# Importance of quantifying the number of viral reads in metagenomic sequencing of environmental samples from the Huanan Seafood Market

**DOI:** 10.1093/ve/vead089

**Published:** 2023-12-30

**Authors:** Jesse D Bloom

**Affiliations:** Fred Hutchinson Cancer Center, Howard Hughes Medical Institute, Seattle, Washington 98109, USA

**Keywords:** huanan seafood market, SARS-CoV-2 metagenomics, COVID-19 origins, raccoon dog

## Abstract

In March 2023, the Chinese CDC publicly released raw metagenomic sequencing data for environmental samples collected in early 2020 from the Huanan Seafood Market. Prior to that data release, some scientists had suggested that these samples could be informative for establishing if animals such as raccoon dogs had been infected with severe acute respiratory syndrome virus 2 (SARS-CoV-2). However, no one had analyzed how much SARS-CoV-2 was actually present in the metagenomic sequencing data. After the raw data became available, I fully analyzed the abundance of both viral and animal genetic material in the samples. That analysis, which was published in *Virus Evolution*, found that the SARS-CoV-2 content of most samples was very low and that the abundance of SARS-CoV-2 was most strongly associated with animals such as largemouth bass that are not plausible candidates for having been infected. Based on these results, I concluded that the metagenomic content of the samples was not informative for determining if any non-human animals in the market had been infected with SARS-CoV-2. One of the authors of an earlier study of these samples, Florence Débarre, recently submitted a response to my paper. Here, I reply in turn to explain why it is important to quantify the abundance of viral material before drawing conclusions from metagenomic sequencing. I also report new analyses of other animal coronaviruses in the samples and show that material from some other animal coronaviruses is much more abundant than SARS-CoV-2 in samples collected on the date when most wildlife stall sampling was performed. I further show that material from some of these animal coronaviruses is associated with the animals they probably infect but that no such association exists for SARS-CoV-2. Overall, these new analyses further emphasize the importance of quantifying the actual amount of viral material in metagenomic samples and underscore why the environmental samples from the Huanan Seafood Market are not informative for determining if any non-human animals were infected with SARS-CoV-2.

## 1. Introduction

In 2022, the Chinese Centers for Disease Control (CDC) posted a pre-print describing environmental samples they collected from the Huanan Seafood Market starting in January 2020 (Liu et al. 2022). That pre-print provided some information about where these samples were collected, as well as a classification made by the Chinese CDC of whether the samples were ‘positive’ for SARS-CoV-2. However, the Chinese CDC did not make available the raw sequencing data or quantitative information about the amount of SARS-CoV-2. This lack of data made it impossible to address all the relevant questions about the environmental samples, and a number of scientists (including myself) commented on social media or in the scientific media that it would be useful if the underlying raw sequence data were made available (Cohen 2022a; Cohen 2022b).

In March 2023, media articles in venues like *The Atlantic* and *New York Times* announced that a team of scientists had obtained the raw sequence data from some Huanan Seafood Market samples and that some samples contained both genetic material from SARS-CoV-2 and animals such as raccoon dogs (Mueller 2023; Wu 2023). These media articles were not accompanied by any actual scientific paper or data. This fact put me and other scientists in an awkward situation: for instance, I was contacted by reporters asking for my thoughts and had to reply that I could not provide an informed comment since I lacked access to the data or any scientific analysis of the data.

Later in March 2023, the team of scientists who had obtained the data posted a scientific report (Crits-Christoph et al. 2023a). This report was more nuanced than the media articles that preceded it and focused largely on analyzing the animal genetic material in samples that the Chinese CDC had called as positive for SARS-CoV-2. Importantly, this report did *not* claim that the samples proved the existence of infected animals. However, it did emphasize that some samples called positive by the Chinese CDC contained genetic material from species such as raccoon dogs that have been hypothesized as potential intermediate hosts for SARS-CoV-2. However, due to a controversy involving the Global Initiative on Sharing All Influenza Data database, Crits-Christoph et al. (2023a) also did not make available the raw sequence data on which their conclusions were based.

After reading the report by Crits-Christoph et al. (2023a), I became concerned that both it and the earlier 2022 Chinese CDC pre-print had overlooked an important question: how much SARS-CoV-2 was actually present in the environmental samples that were being called ‘positive’? The finding of any non-zero amount of SARS-CoV-2 establishes the presence of the virus at the market (or sequencing facility), but this fact has never been in dispute—it has been known since the end of 2019 that there were SARS-CoV-2-infected humans at the market (ProMED 2019). The Chinese CDC samples are *environmental* samples, which means that they were collected from surfaces rather than any specific animal or human. It is therefore unsurprising that some samples might contain some SARS-CoV-2 material, since there had been dozens of infected humans in the market by the time the samples were collected in January 2020 (Huang et al. 2020). In order for the samples to inform about whether an animal was infected, it would be necessary to find an association between genetic material from the virus and the animal species in question. For instance, if the amount of SARS-CoV-2 material was strongly associated with the amount of material from raccoon dogs, that could suggest that raccoon dogs were infected. On the other hand, if the amount of SARS-CoV-2 was more associated with material from animals that are not infectable (say largemouth bass), then that would simply suggest that SARS-CoV-2 was spread widely around the market environment by January 2020. In the latter case, the content of the samples would not be informative as to whether any animal was infected. However, even after the posting of the report by Crits-Christoph et al. (2023a), I was not able to address this question because the raw sequencing data were still not publicly available.

At the very end of March 2023, the Chinese CDC finally made the raw sequencing data available (Liu et al. 2023a; Liu et al. 2023b). At that point, I began my own study to quantify the SARS-CoV-2 content of the samples and the association between the amount of SARS-CoV-2 and material from different animal species. Because I wanted to avoid the problems with data availability associated with earlier discussion of this topic, I created a fully reproducible computational pipeline for my analysis (https://github.com/jbloom/Huanan_market_samples). I completed my analysis by the end of April and posted it as a pre-print on *bioRxiv* (Bloom 2023a); it was subsequently published after peer review with minor revisions in *Virus Evolution* (Bloom 2023b). The purpose of the current paper is not to fully recap that analysis, so I encourage readers to read Bloom (2023b) (the original paper) as background. But my key conclusions are as follows:

1. The amount of SARS-CoV-2 in most environmental samples was low, and the Chinese CDC had required only a single SARS-CoV-2 sequence read (usually out of hundreds of millions of reads total) to call a sample as ‘positive’.
2. The amount of SARS-CoV-2 in samples that contained appreciable material from non-human animal species that might be susceptible to SARS-CoV-2 was uniformly low, with the ‘positive’ sample containing abundant material from raccoon dog that had been the focus of the initial media attention (Mueller 2023; Wu 2023), only containing one SARS-CoV-2 read out of ~200,000,000 total reads.
3. To the extent that the number of SARS-CoV-2 reads was associated with material from any animals, the strongest associations were with species such as largemouth bass that are not plausible candidates for having been infected.
4. Due to these facts, I concluded that for these environmental samples, *‘co-mingling of animal and viral genetic material is unlikely to reliably indicate whether any animals were infected by SARS-CoV-2’* (Bloom 2023b).

Recently, a member of the original team that published the report by Crits-Christoph et al. (2023a) on the metagenomic content of the samples, Flo Débarre, submitted a response to my paper (Débarre 2024). In this response, she questions the rationale for my analysis. This paper provides my reply to her response. It also includes new analyses that show that some other animal coronaviruses are associated with the animals they infect in the environmental samples, but that this is not the case for SARS-CoV-2.

## 2. Results

### 2.1. It is important to analyze the absolute quantity of viral material in metagenomic samples

In any metagenomic analysis, it is important to quantify the *abundance* as well as the presence–absence of reads mapping to each species or virus of interest. Environmental samples from crowded markets will generally contain genetic material of many viruses and species. Interpreting metagenomic sequencing therefore requires analyzing how many sequencing reads map to different potential sources of genetic material.

In fact, abundance quantification is done for the animal species in the original report by Crits-Christoph et al. (2023a), which shows pie charts of what fraction of reads map to each species (pie charts are a form of abundance quantification) and even imposes an abundance threshold on how many reads must map to an animal species to be considered meaningful. Specifically, the Methods of Crits-Christoph et al. (2023a) says *‘A minimum of 400 covered bases (which corresponds to ~10 43-bp reads) was required to mark a species as positive in a given sequencing run of a sample’.* This is a sensible requirement. If a sample with hundreds of millions reads has just a handful of reads mapping to a particular animal species, it probably indicates just trace material from the market environment, not true deposition of that sample by that animal.

However, neither the original Chinese CDC pre-print (Liu et al. 2022) nor Crits-Christoph et al. (2023a) performed a similar abundance quantification for SARS-CoV-2 in the environmental samples. My study was the first to perform such a quantification—and it found that most of the samples that the Chinese CDC had classified as ‘positive’ had very low amounts of SARS-CoV-2 (Bloom 2023b).

For instance, the Q61 sample that was analyzed in detail in Crits-Christoph et al. (2023a) because it contained abundant raccoon dog material and was classified as SARS-CoV-2 ‘positive’ by the Chinese CDC turned out to contain only 1 of ~200,000,000 reads mapping to SARS-CoV-2 (Bloom 2023b). This SARS-CoV-2 content is substantially lower than that of some other environmental samples from the market that have most of their animal genetic material derived from species like largemouth bass that certainly were never infected with SARS-CoV-2 (Bloom 2023b). A sample could end up with 1 of ~200,000,000 reads that mapped to SARS-CoV-2 for a whole suite of possible reasons including deposition of SARS-CoV-2 from an infected human in the market, contamination from a human during sample collection, or index-hopping during sequencing. Presumably, it is one of these reasons that caused thousands of SARS-CoV-2 reads to be found in samples where most animal genetic material is from species like largemouth bass or catfish (Bloom 2023b), so it is certainly possible that these reasons could also explain the presence of just a handful of SARS-CoV-2 reads in samples where most animal genetic material is from species like raccoon dogs.

### 2.2. SARS-CoV-2 is at much lower abundance than some other animal coronaviruses in samples collected on 12 January 2020

Débarre (2024) suggests that perhaps the reason that the SARS-CoV-2 content is so low in the samples with abundant material from species such as raccoon dogs is that many samples from the wildlife stalls were collected on 12 January 2020, which is later than the collection date 1 January 2020 of some other samples. She points out that this is a confounding factor: for instance, maybe the viral RNA in the wildlife stall had mostly degraded before the samples were collected. I certainly agree that the variation in sampling date a confounder and discuss this in some detail my paper. For instance, Bloom (2023b) provides interactive versions of figures that allow stratification of the results by sampling date.

However, even given the sampling date, the SARS-CoV-2 content is very low in the samples collected from the wildlife stalls on 12 January 2020. To demonstrate this fact, here I further analyze the metagenomic sequencing based on the observation originally made by Massey (2023) and subsequently elaborated by Crits-Christoph et al. (2023b) that the environmental samples collected on 12 January 2020 also contain material from other animal coronaviruses. These other coronaviruses can establish a baseline for how much viral RNA might still be present by 12 January 2020.

**Figure 1.**
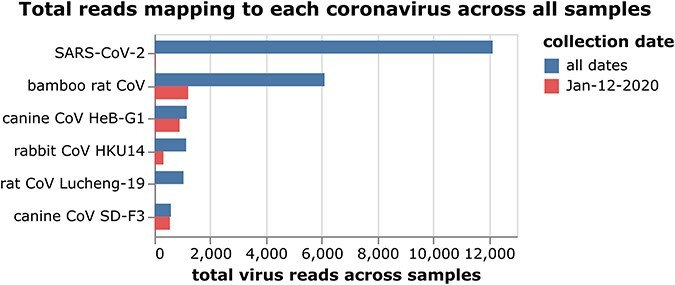
Total viral reads mapping to each coronavirus across samples from all collection dates, or only the date 12 January 2020 when most wildlife-stall sampling was performed. This figure shows only coronaviruses with at least 500 mapped reads across all samples. See https://jbloom.github.io/Huanan_market_samples_addtl_analysis/viral_counts.html for an interactive figure that allows one to mouseover bars for details and show additional collection dates.

Five animal coronaviruses in addition to SARS-CoV-2 had at least 500 sequencing reads across all samples from all collection dates (Figure 1). Four of these coronaviruses (bamboo rat CoV, canine CoV HeB-G1, rabbit CoV HKU14, and canine CoV SD-F3) have a substantial amount (at least a quarter) of their reads from samples collected on the 12-January-2020 wildlife-stall collection date (Figure 1). The other two coronaviruses (SARS-CoV-2 and rat CoV Lucheng-19) have only a very small fraction of their reads from samples from the 12-January-2020 wildlife-stall collection date.

**Figure 2.**
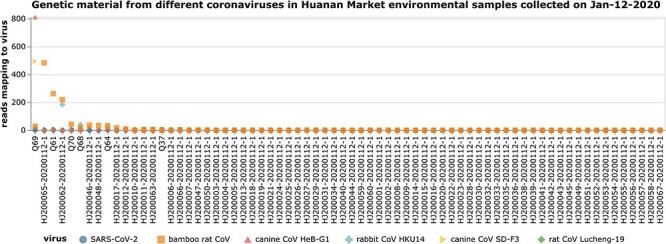
Number of deep sequencing reads mapping to each of six coronaviruses among all metagenomically sequenced samples collected from the Huanan Seafood Market on 12 January 2020, which was the date of most wildlife-stall sampling. The plot shows per-sample read counts for SARS-CoV-2 and all animal coronaviruses with at least 500 total read counts across all collection dates. For four of the five animal coronaviruses (bamboo rat CoV, canine CoV HeB-G1, rabbit CoV HKU14, and canine CoV SD-F3), there is at least one 12-January-2020 sample that has hundreds of mapped viral reads. However, no 12-January-2020 sample has more than seven reads mapping to SARS-CoV-2 or more than one read mapping to rat CoV Lucheng-19. See https://jbloom.github.io/Huanan_market_samples_addtl_analysis/viral_reads_per_sample.html for an interactive figure that allows one to mouse over points for details, change the *y*-axis from a linear to log10 scale, and make other interactive changes to the plot display.

For four of the five non-SARS-CoV-2 animal coronaviruses, one or more 12-January-2020 environmental sample has at least several hundred viral reads (Figure 2). The read counts for these four animal coronaviruses are asymmetrically distributed, with a few samples appearing to be truly ‘positive’ (having hundreds of reads) and most samples appearing to be ‘negative’ (having little or no viral reads) (Figure 2). In contrast, no samples from 12-January-2020 have even ten SARS-CoV-2 reads or more than one rat CoV Lucheng-19 read (Figure 2). These results do not disprove the possibility of SARS-CoV-2-infected animals—there are possible confounders even when the analysis is restricted to just the 12-January-2020 samples. However, Figure 2 does show that there is an appreciable amount of coronavirus RNA present in some environmental samples collected on 12-January-2020—this RNA just comes from other animal coronaviruses, not SARS-CoV-2.

### 2.3. There are associations between viral and animal genetic material for the some animal coronaviruses, but not for SARS-CoV-2

Débarre (2024) suggests that an analysis of the association between the amount of viral and animal genetic material would never be expected to yield meaningful results (she says this is an analysis ‘we knew would fail’). To assess this claim, here I analyze the joint viral and animal content of all samples collected on 12 January 2020 for SARS-CoV-2 and the five animal coronaviruses with at least 500 total reads across all collection dates.

**Figure 3.**
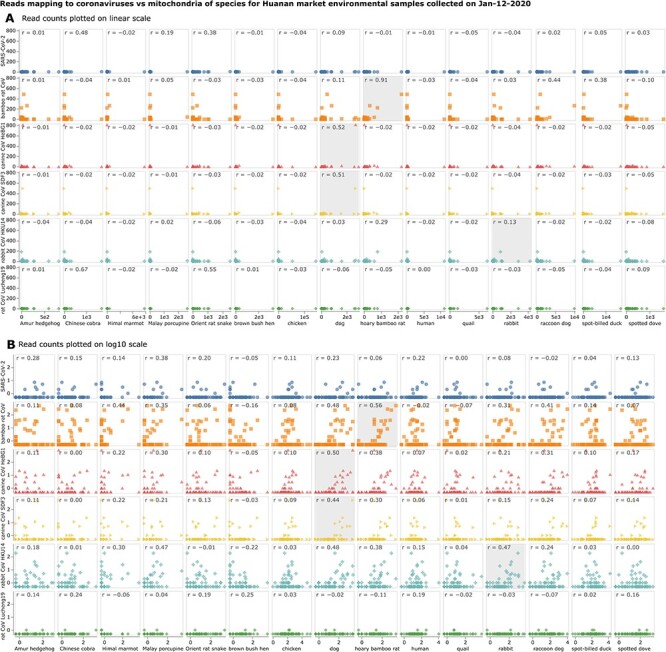
Association between the number of reads mapping to the mitochondrial genome of each animal versus each coronavirus shown on a **(A)** linear or **(B)** log10 scale for all metagenomically sequenced samples collected from the Huanan Seafood Market on 12 January 2020. Each point represents the number of animal mitochondrial and viral reads for a different sample. The numbers in the upper left of each panel give the Pearson correlation (*r*). The plot includes all species that contribute at least 20 per cent of the chordate mitochondrial genomic material to at least one sample with at least one read from any of the indicated coronaviruses. For the animal coronaviruses, the panel corresponding to the animal species that is known to be infected by that virus is shaded. For the log10 scales, values of zero are plotted as half the minimum observed non-zero value, since zero itself cannot be plotted on a log scale. See https://jbloom.github.io/Huanan_market_samples_addtl_analysis/viral_subset_species_corr.html for an interactive version of this plot, and https://jbloom.github.io/Huanan_market_samples_addtl_analysis/viral_all_species_corr.html for an interactive plot that shows all chordate species that meet the criteria described in Bloom (2023b).

For four of the animal coronaviruses (bamboo rat CoV, canine CoV HeB-G1, rabbit CoV HKU14, and canine CoV SD-F3), the samples that have a substantial number of viral reads also have a high content of genetic material from the animal known to be infected by that virus (Figure 3). For instance, the samples with the most bamboo rat coronavirus reads all have abundant bamboo rat genetic material and likewise for the HeB-G1 or SD-F3 canine coronaviruses and the rabbit CoV HKU14 (see shaded boxes in Figure 3). In fact, for three of these four animal coronaviruses, the strongest correlation between the number of viral and animal reads is for the animal species known to be infected with the virus (Figure 3). In addition, for all four of these animal coronaviruses, the sample with the most viral reads has the largest fraction of its mitochondrial genetic material from the animal known to be infected with that virus (to see this, mouseover points in the interactive plots at https://jbloom.github.io/Huanan_market_samples_addtl_analysis/viral_reads_per_sample.html or https://jbloom.github.io/Huanan_market_samples_addtl_analysis/viral_subset_species_corr.html). In contrast, the quantitative content of SARS-CoV-2 and rat CoV Lucheng-19 is low in all 12-January-2020 samples, and to the extent that there are associations with animal genetic material, they are weak and contingent on how the data are quantified (e.g. linear versus log scale).

The results in Figure 3 therefore show that analyses of the associations between the amount of viral and animal genetic material are not always destined to ‘fail’. For the animal coronaviruses with the most material in the 12-January-2020 samples, there is often an association between the virus and the animal species infected by that virus. Admittedly, these correlations are driven by just a few points and so are not sufficient to establish that one should always expect such a correlation between a virus and its true host in such samples. Therefore, the fact that SARS-CoV-2 does not show an association with a plausible host animal species does not disprove the possibility of SARS-CoV-2-infected animals. But if SARS-CoV-2 had shown similar patterns to some of the other animal coronaviruses in the 12-January-2020 samples, that would have provided moderate circumstantial evidence in favor of SARS-CoV-2-infected animals—but this is not the case.

So contrary to the claim of Débarre (2024), analyses of associations between viral and animal genetic material are not always destined to ‘fail’. For some animal coronaviruses, such an analysis actually does reveal an association between material from the virus and the animal it is known to infect among the 12-January-2020 samples. The lack of such an association for SARS-CoV-2 is not a ‘failure’, but rather a real scientific result that should be reported—even if, as I say in Bloom (2023b), the evidence remains sufficiently equivocal that all we can really conclude is that the environmental samples are insufficient to establish whether any animals were infected.

### 2.4. None of the conclusions in my paper depend on the statistical significance of correlation coefficients or arbitrary thresholds

Débarre (2024) criticizes the use of correlation coefficients to quantify the associations between viral and genetic material. However, I never make any statistical claims based on these coefficients, but simply use them as a shorthand to quantify associations that are also shown by visual display of all data. Specifically, in both my original paper (Bloom 2023b) and Figure 3 here, I provide scatter plots that fully show the data, as well as interactive scatter plots linked in the figure legends.

Scatter plots are a good way to show all the data without any need for choosing arbitrary thresholds about how many reads are needed to call a sample ‘positive’ for a given virus or animal. I encourage the reader to just spend a few minutes looking at these scatter plots. For instance, no statistics are needed to see that Figure 3 shows possibly meaningful associations between viral and animal genetic material for some animal coronaviruses, but not for SARS-CoV-2. Correlation coefficients can be a useful shorthand to summarize associations in scatter plots, but the same conclusions are apparent from just visually inspecting the data. Crucially, these scatter plots do not require choosing any arbitrary threshold for how much viral or animal material must be found in a specific sample for it to be called ‘positive’, but instead allow you to see the full span of the data.

Furthermore, I never claimed that correlation coefficients proved any particular conclusion to be true—instead, I pointed out that the mostly weak and nonsensical correlations between SARS-CoV-2 and animal genetic material simply indicated that the environmental samples were inconclusive as to the existence of SARS-CoV-2-infected animals. Recall that the abstract of Bloom (2023b) states that my analysis shows that *‘co-mingling of animal and viral genetic material is unlikely to reliably indicate whether any animals were infected by SARS-CoV-2’*.

### 2.5. Minor corrections

Débarre (2024) suggests two minor errors in my paper.

**Figure 4.**
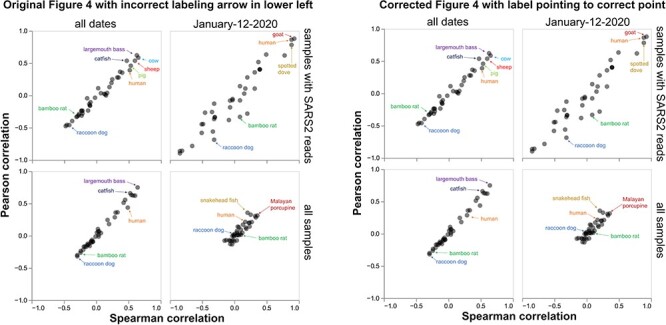
A minor labeling error in the fourth figure of Bloom (2023b) does not affect any of the conclusions. This figure was used to conclude that the strongest correlations between SARS-CoV-2 and animal genetic material was for species (e.g. largemouth bass) that were not plausible candidates for being infected. Débarre (2024) correctly noted that in the lower-left panel of original figure in Bloom (2023b) (reprinted above at left), the arrow for human indicates the wrong point. This is fixed in the lower-left panel of the corrected figure (reprinted above at right). Note that this labeling error does not affect any of the conclusions since it remains true that the strongest correlations are with species (e.g. largemouth bass) that are not plausible candidates for having been infected. The error arose when I was manually labeling points for the figures in Bloom (2023b); the correlations themselves are all computed correctly and have always been correct in the interactive version of the plot (see https://jbloom.github.io/Huanan_market_samples/overall_corr.html).

First, she notes that I made a minor error when manually labeling one of the points in the lower-left panel of the fourth figure of Bloom (2023b). I thank her for noting this minor error and have submitted a correction to *Virus Evolution* to fix the label (see Figure 4 for the original and corrected figure). Note, however, that the mis-labeling had no consequence for the conclusions of my analysis: my conclusion was that the strongest correlations are with species that are not plausible candidates for having been infected, and this conclusion remains true after correcting the minor labeling error (Figure 4).

Second, Débarre (2024) suggests that my analysis incorrectly excluded two metagenomic samples, F13 and F54. I disagree that this is an error. The Chinese CDC sequenced samples in several ways: most were just metagenomically sequenced, but a small number were sequenced by other methods designed to enrich for viral RNA. Samples that were not subject to pure metagenomic sequencing should be excluded, as enrichment for viral RNA will bias the results. as I clearly state in Bloom (2023b), I restricted my analysis just to the samples described by the Chinese CDC as ‘RNA sequencing of total nucleic acids from environmental swabs for metagenomics’ and exclude the handful of samples that were described as ‘RNA sequencing of total nucleic acids from environmental swabs for metagenomic analysis and viral whole-genome assembly’ or ‘SARS-CoV-2 Amplicon based SARS-CoV-2 whole genome sequencing for cell supernatants’. The data sheet for the samples clearly describes all experiments for samples F13 and F54 as ‘RNA sequencing of total nucleic acids from environmental swabs for metagenomic analysis and viral whole-genome assembly’. Specifically, see the ‘Experiment’ tab of the Excel datasheet CRA010170.xlsx at https://ngdc.cncb.ac.cn/gsa/browse/CRA010170, which reports the library construction method for sample names Env_0313 and Env_0354 (which are the Chinese CDC identifiers for F13 and F54). Therefore, while I agree (as discussed in the ‘Limitations of this study’ section of Bloom (2023b)) that the information provided on sample processing by the Chinese CDC is limited, samples F13 and F54 are properly excluded by the reasonable and consistent criteria of only analyzing purely metagenomically sequenced samples according to the experiment descriptions provided by the Chinese CDC in their metadata sheet.

Furthermore, I note that even if one were to include samples F13 and F54, that would in no way change the conclusion of Bloom (2023b) that the SARS-CoV-2 content of the samples is not correlated with genetic material from any potentially susceptible non-human animal. As can be seen from analyzing the table at https://github.com/jbloom/Huanan_market_samples/blob/main/results/aggregated_counts/mito_composition_by_sample.csv, the most abundant species for these two samples are human, chicken, and spot-billed duck (for sample F13) and human, largemouth bass, and carp (for sample F54). Neither of these samples contain any reads mapping to the mitochondria of potentially susceptible wild animals such as raccoon dogs or bamboo rats.

### 2.6. Other arguments about the role of the market

Débarre (2024) also makes a number of other arguments about the role of the market; most of these arguments are outside the scope of my original study (Bloom 2023b), so I will not try to address them all here. However, I will note that some of these arguments have to do with the spatial distribution of samples called ‘positive’ by the Chinese CDC. But as described here and in Bloom (2023b), the incontrovertible fact is that there just are not very many SARS-CoV-2 reads in the metagenomic sequencing of any of the wildlife-stall samples—and the tiny amounts that are found are much less than the SARS-CoV-2 content of samples collected on other dates from other regions of the market and also much less than the amount of several other animal coronaviruses in samples collected from the wildlife stalls on 12 January 2020. The fact that these ‘positive’ samples contain very little SARS-CoV-2 represents a significant caveat for any analysis of their spatial distribution.

## 3. Discussion

In the first results section of her paper, Débarre (2024) writes that I was doing an analysis that she and her co-authors ‘knew would fail’. I respectfully disagree. As I have explained earlier, a crucial part of any metagenomics analysis is quantifying the abundance of reads mapping to each species or virus. Bloom (2023b) was the first study to quantify the number of SARS-CoV-2 reads in the metagenomic sequencing of the environmental samples and revealed that the SARS-CoV-2 content is extremely low in the samples that contained appreciable material from non-human animals that could plausibly have been infected with SARS-CoV-2. This result does not mean that the analysis ‘failed’—it just means that the metagenomic content of the samples is not able to establish whether any animals were infected by SARS-CoV-2. Such a result is a valid finding that is important to report.

Furthermore, the new analyses described in Figures 2 and 3 show that the low SARS-CoV-2 content and lack of associations with animal genetic material are not an inherent feature of the samples collected on the 12-January-2020 wildlife-stall focused sampling date. In fact, some of the samples collected on that date contain a substantial number of reads mapping to other animal coronaviruses. The abundance of material from some of these other animal coronaviruses does associate with material from the animals those viruses are known to infect. Therefore, it is possible for the metagenomic content of environmental samples to provide moderate circumstantial evidence for coronavirus infection of animals. However, the metagenomic samples from the Huanan Seafood Market do not provide any such evidence for SARS-CoV-2. As I emphasize in Bloom (2023b), this fact does not disprove the possibility of SARS-CoV-2-infected animals—there remain possible confounders that could lead to a lack of association between reads from a virus and an animal it infects in these metagenomic samples. But the new analyses presented in this paper further support the contention of my original study that the Huanan Seafood Market environmental samples are insufficient to reliably determine whether any animals were infected by SARS-CoV-2.

## 4. Limitations of this study

The final section (section 4) of Bloom (2023b) discusses limitations of any study of the metagenomic content of the Chinese CDC’s environmental samples from the Huanan Seafood Market. All the limitations discussed in that section of Bloom (2023b) also apply to the current study, so I encourage the reader to re-read them. As I did in my original study, I have provided a fully reproducible computational pipeline (https://github.com/jbloom/Huanan_market_samples_addtl_analysis) as well as interactive plots (https://jbloom.github.io/Huanan_market_samples_addtl_analysis) for all new analyses reported in the current paper.

## 5. Methods

The figures shown here were created by extending the analysis in Bloom (2023b) to also analyze reads mapping to animal coronaviruses in addition to SARS-CoV-2. See https://github.com/jbloom/Huanan_market_samples_addtl_analysis for a fully reproducible computational pipeline that performs the analysis. See https://jbloom.github.io/Huanan_market_samples_addtl_analysis for interactive versions of all plots that allow mousing over points for details and other options.

Briefly, I downloaded from the National Genomics Data Center (NGDC) the FASTQ files for all the sequencing runs described in the Chinese CDC analysis (Liu et al. 2023b) (see the list of FASTQ files at https://raw.githubusercontent.com/jbloom/Huanan_market_samples/main/results/metadata/merged_metadata.csv). I pre-processed these FASTQ files with fastp (Chen et al. 2018).

I then constructed a reference database consisting of SARS-CoV-2 and the animal coronaviruses listed in Table S17 of Crits-Christoph et al. (2023b), which gives the following set of Genbank accessions and coronaviruses:

- NC_017083.1: rabbit CoV HKU14
- NC_026011.1: rat CoV HKU24
- NC_032730.1: rat CoV Lucheng-19
- NC_039208.1: porcine CoV HKU15
- NC_045512.2: SARS-CoV-2
- OM451122.1: canine CoV SD-F3
- OM451123.1: canine CoV HeB-G1
- OM451212.1: hedgehog CoV HKU31 HeN-F3
- OM451213.1: hedgehog CoV HKU31 HeB-MO1
- OQ297694.1: bamboo rat CoV

I trimmed the 3’ polyA tails from these genomes, then aligned the FASTQ files to them using minimap2 (Li 2018) in its short-read (sr) mode, and filtered the resulting binary alignment and map (BAM) files for reads with mapping qualities of at least four. I then computed the number of reads and number of covered bases for each coronavirus genome using coverM (https://github.com/wwood/CoverM) with a minimum read aligned length of 40, a contig end exclusion of 100 nucleotides, and a minimum read percent identity of 95 per cent. See https://github.com/jbloom/Huanan_market_samples_addtl_analysis/blob/main/results/viral_alignment_counts_and_coverage/aggregate_counts_and_coverage.csv for statistics on the number of reads mapping to each virus for each accession.

I then merged these viral read counts with the number of reads mapping to the mitochondrial genome of each animal species as previously computed in Bloom (2023b), aggregating counts for each sample and only retaining runs described by the Chinese CDC as ‘RNA sequencing of total nucleic acids from environmental swabs for metagenomics’. See https://github.com/jbloom/Huanan_market_samples_addtl_analysis/blob/main/results/merged_viral_and_mito_counts.csv for these merged viral and animal mitochondrial genome read counts.

I then calculated the total number of viral reads mapping to each coronavirus and only retained the six coronaviruses (those shown in Figure 1) with at least 500 reads across all samples.

For Figures 2 and 3 , I subsetted to just the samples collected on 12 January 12 2020, which was the date of most intensive wildlife-stall sampling. This subsetting to just one date ensures that all the samples being analyzed have had the same amount of time elapsed prior to collection, which should help ameliorate any biases caused by degradation of viral RNA. The scatter plots of viral versus animal mitochondrial read counts in Figure 3 show just species that contributed at least 20 per cent of the animal mitochondrial reads in a sample with at least one viral read. See https://jbloom.github.io/Huanan_market_samples_addtl_analysis/viral_all_species_corr.html for a much larger plot that shows all chordate species that met the criteria described in Bloom (2023b).

For the plots, when read counts are shown on a log scale, values of zero are plotted as half the minimum non-zero value since zero itself cannot be plotted on a log scale.
